# Acidic fibroblast growth factor attenuates type 2 diabetes-induced demyelination via suppressing oxidative stress damage

**DOI:** 10.1038/s41419-021-03407-2

**Published:** 2021-01-21

**Authors:** Rui Li, Beini Wang, Chengbiao Wu, Duohui Li, Yanqing Wu, Libing Ye, Luxia Ye, Xiongjian Chen, Peifeng Li, Yuan Yuan, Hongyu Zhang, Ling Xie, Xiaokun Li, Jian Xiao, Jian Wang

**Affiliations:** 1 Department of Hand Surgery and Peripheral Neurosurgery, The First Affiliated Hospital and School of Pharmaceutical Sciences, Wenzhou Medical University, 325000 Wenzhou, Zhejiang China; 2 Research Center, Affiliated Xiangshang Hospital, Wenzhou Medical University, 315700 Ningbo, Zhejiang China; 3 School of Chemistry, Sun Yat-sen University, 510275 Guangzhou, Guangdong China

**Keywords:** Growth factor signalling, Molecular neuroscience

## Abstract

Prolonged type 2 diabetes mellitus (T2DM) produces a common complication, peripheral neuropathy, which is accompanied by nerve fiber disorder, axon atrophy, and demyelination. Growing evidence has characterized the beneficial effects of acidic fibroblast growth factor (aFGF) and shown that it relieves hyperglycemia, increases insulin sensitivity, and ameliorates neuropathic impairment. However, there is scarce evidence on the role of aFGF on remodeling of aberrant myelin under hyperglycemia condition. Presently, we observed that the expression of aFGF was rapidly decreased in a db/db T2DM mouse model. Administration of exogenous aFGF was sufficient to block acute demyelination and nerve fiber disorganization. Furthermore, this strong anti-demyelinating effect was most likely dominated by an aFGF-mediated increase of Schwann cell (SC) proliferation and migration as well as suppression of its apoptosis. Mechanistically, the beneficial biological effects of aFGF on SC behavior and abnormal myelin morphology were likely due to the inhibition of hyperglycemia-induced oxidative stress activation, which was most likely activated by kelch-like ECH-associated protein 1 (Keap1)/nuclear factor erythroid-derived-like 2 (Nrf2) signaling. Thus, this evidence indicates that aFGF is a promising protective agent for relieving myelin pathology through countering oxidative stress signaling cascades under diabetic conditions.

## Introduction

Type 2 diabetes mellitus (T2DM), a clinical chronic disease worldwide, has become an epidemic disease accompanying insulin resistance and insulin secretion defect^1,2^. Statistically, the annual expenditure for T2DM management is approximately $825 billion around the world every year, and T2DM produces an enormous financial burden for the health care system^3,4^. Unfortunately, there are no effective therapeutic strategies to cure this chronic disease, except for rigorous glucose control^5^. Long-term hyperglycemia in the peripheral nervous system (PNS) causes nerve damage and cytotoxicity, which leads to myelin sheath vacuolar degeneration and the generation of numerous onion bulb-shaped formations. If these pathological changes continue throughout T2DM progression, the impaired nerve fibers undergo segmental demyelination, eventually leading to diabetic peripheral neuropathy (DPN). Experimental and clinical studies have demonstrated that patients with demyelinating neuropathy have a higher risk of developing sensory deficits and muscle weakness, as well as foot ulceration^6–9^. Thus, preventing demyelination progression in T2DM is a critical issue for ameliorating abnormal nerve function.

As supporting gliocytes in the PNS, Schwann cells (SCs) produce consecutive layers of the plasma membrane that enwrap axons; these layers form myelin sheaths as they mature. During the process of remyelination, SCs are pivotal for regulating myelin thickness, maintaining the normal conduction of electrical impulses and providing trophic support for axon regeneration^10,11^. Emerging evidence suggests that SCs are crucial to modulate the pathogenesis of DPN and chronic hyperglycemia can induce SC dysfunction in diabetic humans and felines^12–15^. Given the pivotal role of SCs in myelin maintenance and nerve regeneration, enhancing the proliferation and migration of SCs and restraining their apoptosis may be an ideal therapy to attenuate demyelination in acute or chronic diabetes.

Acidic fibroblast growth factor (aFGF), also known as FGF1, is an autocrine/paracrine regulator that controls cell proliferation, migration, and survival through binding its high-affinity receptors (FGFR1-4)^16^. The application of recombinant human aFGF to facilitate wound or burn repair in diabetic patients has achieved excellent clinical therapeutic effect. Furthermore, previous investigations confirmed that aFGF is expressed extensively in the mammalian nervous system and the progressive decrease of aFGF is identified in the peripheral blood of patients with T2DM^17,18^. aFGF is also shown to have powerful neuroprotective and neuroregenerative effects that facilitated neurite outgrowth, stimulated motor and sensory axon regeneration, and prevented neuronal death postaxotomy^19–21^. Importantly, aFGF has emerged as a promising solution to relieve diabetic hyperglycemia and enhance insulin sensitization^22,23^. Conversely, this antidiabetic effect was abolished when the aFGF gene was knocked out^24,25^. Based on the important role of aFGF in regulating glucose uptake, we speculate that aFGF protects SCs from high-glucose-induced excessive apoptosis, ultimately contributing to myelin regeneration and remodeling after pathological changes to the peripheral nerve under diabetic condition. However, we still do not understand the mechanism underlying aFGF ameliorates demyelination.

Chronic hyperglycemia in diabetes triggers various pathological changes, including advanced glycation, polyol pathway activity, and superoxide-induced free radical formation^26^. Recently, a growing body of studies has suggested that oxidative stress is a major hallmark of T2DM and its associated complications^27,28^. Under hyperglycemic condition, the production of reactive oxygen species (ROS) is higher than that of the formation of antioxidants. This disruption of redox homeostasis elicits the peroxidation of proteins, lipids, and nucleic acids and further triggers the excessive activation of oxidative stress, which leads to insulin resistance, metabolic dysfunction, and even irreversible nerve cell death. Previous studies had demonstrated that chronic T2DM-induced excessive oxidative stress increased the vulnerability of peripheral nerves to diabetic neuropathy and cell death^29,30^. Furthermore, an upregulation of oxidative stress in diabetic rodents was found to increase the misfolding of myelin-related proteins and alter myelin structure, which might have important implications for the demyelination process^31^. Additionally, the disruption of SC metabolism under diabetic condition induced the rapid and robust accumulation of toxic oxygen free radicals and peroxides, which severely damaged axon and impaired vascular function^32,33^. Hence, decreasing the level of oxidative damage under diabetic condition is believed to be an important factor for reducing neuron demyelination.

Nrf2 acts as an important transcription factor for defending against oxidative damage and toxic insults^34^. A line of evidence suggests that the Nrf2-mediated cellular redox balance is regulated by Keap1^35–37^. Importantly, Keap1/Nrf2 signaling can drive cell proliferation and inhibit apoptosis through increasing various enzymatic antioxidants during diabetic neuropathy^38^. Thus, Nrf2/Keap1 signaling is an important regulatory pathway involved in defending against oxidative damage-induced neurodegenerative disease.

In the present study, we tried to explore whether aFGF has a significant effect on attenuating demyelination in T2DM and reveal its potential regulatory mechanism. Our findings indicated that aFGF ameliorates demyelination and upregulates myelin-related proteins and genes in chronic T2DM, and these effects were likely regulated by facilitating SC proliferation and migration and inhibiting its apoptosis. Unexpectedly, the anti-demyelinating and glial protective effects were correlated with aFGF-mediated activation of Keap1/Nrf2 pathway, then further inhibiting excessive oxidative stress within SCs. These findings suggest that aFGF has therapeutic potential for preventing demyelination in patients with diabetes.

## Materials and methods

### Reagents and antibodies

aFGF was provided by the Key Laboratory of Biotechnology Pharmaceutical Engineering at Wenzhou Medical University, Wenzhou, Zhejiang, China. For the other reagents: hematoxylin and eosin (H&E, Boster, AR1180), neutral resin (Solarbio, Cat#G8590), phosphate buffered saline (PBS, Solarbio, P1010), Reactive Oxygen Species Assay Kit (ROS Assay Kit, Beyotime Biotechnology, S0033), bovine serum albumin (BSA, Beyotime Biotechnology, STO23), Dulbecco’s modified Eagle Medium (DMEM, gibico, 11965092), Reverse transcription PCR Kit (RT-PCR Kit, TaKaRa, RR037A), 4’6-diamidino-2-phenylindole-dihydrochloride (DAPI, Beycotime Biotechnology, C1006), Triton X-100 (Beycotime Biotechnology, T8200), TUNEL Apoptosis Detection Kit (Alexa Fluor 488, YE SEN Biotechnology, 40307ES60), Nuclear and Cytoplasmic Protein Extraction Kit (Beyotime Biotechnology, P0028).

The related information of primary antibodies and their concentration used in this study was provided as follows: mouse polyclonal anti-FGF1 (Abcam, ab169748, 1:3000), anti-S100 (Santa Cruz, sc-53438, 1:500), mouse monoclonal anti-MBP (Abcam, ab62631, 1:3000), rabbit polyclonal anti-MPZ (Abcam, ab31851, 1:3000), mouse monoclonal anti-PCNA (Abcam, ab29, 1:500), rabbit monoclonal anti-Ki67 (Abcam,ab92742, 1:5000), rabbit monoclonal anti-Bax (CST, #14796, 1:500), rabbit monoclonal anti-Bcl-2 (CST, #4223, 1:500), rabbit polyclonal anti-Cleaved caspase-3 (Abcam, ab2302, 1:500), rabbit polyclonal anti-SOD2 (Abcam, ab13533, 1:5000), mouse monoclonal anti-HO-1 (Abcam, ab13248, 1:1000), rabbit monoclonal anti-NQO1 (Abcam, ab80588, 1:1000), rabbit monoclonal anti-Keap1 (CST, #8047, 1:1000), rabbit monoclonal anti-Nrf2 (Abcam, ab76026, 1:5000), rabbit polyclonal anti-GAPDH (Millipore, AB2302, 1:10000), rabbit monoclonal anti-Histone H3 (Abcam, ab176842, 1:1000).

### Animal model and drug treatment

Male C57BL/6J mice were bred in the Model Animal Research Center of Nanjing University (Nanjing, China). The process of inducing T2DM model was described previously^39^. Briefly, C57BL/6 male mice, body weight of 20–30 g, were fed a high-fat diet (D12495; Research Diets) containing 10% fat, 1.5% cholesterol, 0.25% sodium cholate, 5% sucrose, and 83.25% basic feed for up to 12 weeks. Whereas, the mice in the control group were fed a standard diet (A500SL, Roles-Bio). Food intake and body weight were measured once a week. It should be emphasized that all the T2DM model animals used in the present study had met the following conditions: the blood glucose concentration ≥16.7 mmol/L and the body’s weight over 20% of the mean value of the control. The protocols for animal care and use were conducted in accordance with the Guide for the Care and Use of Laboratory Animals from the National Institutes of Health. Before the start of the study, rats were allowed to acclimate to the facility for at least one week. Then, the total animals were randomly divided into control, T2DM and T2DM + aFGF groups, each group contained 10 mice. For the latter two groups, the high-fat diet mice were administered either with aFGF (0.5 mg/kg body weight) or equal volume of physiologic saline via intraperitoneal (i.p.) injection every other day for one month. aFGF was dissolved in saline to achieve the working concentration of 20 μg/mL. Later, the mice were euthanized, and sciatic nerve tissues were collected for the various pathology index analysis.

### Ultrastructural observation

The ultrastructure of sciatic nerves in each group was observed under TEM. Briefly, samples were fixed in 2.5% glutaraldehyde for at least 48 h. The tissues were then immersed in 1% osmium tetroxide solution and 1% uranyl acetate for 1 h, respectively. Thereafter, the samples were dehydrated, embedded, and placed in baking box (45 °C) to keep dry for 72 h. Lastly, the dry segments were cut and examined by TEM (H-600; HITACHI, Japan). Quantitative analyses were performed on five animals per group; each group randomly selected 100 myelinated axons from 10 images with the area of 30 × 30 μm^2^ to measure axon diameter and calculate g-ratio using Image J. The g-ratio was obtained by determining the inner axon diameter divided by the outer diameter of the myelin.

### Histological assessment

After perfusion, the sciatic nerves in each group were harvested and fixed in a cold 4% paraformaldehyde at 4 °C for 24 h. Afterward, the tissues were dehydrated in gradient concentrations of alcohol (70%, 80%, 90%, 100%) and embeded in paraffin. Next, the cross-sections of the nerve samples were cut in 8 μm thicknesses for Hematoxylin-eosin (H&E) staining. The images were acquired using a Nikon ECLIPSE 80i (Nikon, Japan).

### Real-time PCR

Firstly, the collecting sciatic nerves were homogenized in TRIzol reagent. Then, total RNA was reverse-transcribed into cDNA using Reverse Transcription Reagents Kit (TaKaRa, RR037A). The real-time PCR was conducted in a 7900HT Fast Real-Time PCR System using SYBR Green PCR Master Mix (Bio-Rad, Hercules, CA, USA). Lastly, the results were expressed as 2^^(–ΔΔCt)^. The primer sequence of Egr2, MBP, MPZ, Pmp22, and Sox10 genes for real-time PCR were listed in Table 1.

**Table 1:** Primers used for RT-PCR in this study.

Gene	Prime sequence	Product size (bp)	Serial number
β-actin	F: GCAAGTGCTTCTAGGCGGACTG	195	NM_001101683.1
	R: CTGCTGTCACCTTCACCGTTCC		
MBP	F: AGTCCGACGAGCTACAGACCATC	106	XM_017338987.1
	R: TACTTGGAGCCGTGCCTCTGG		
MPZ	F: TCATCGAGATGGAGCTACGGAAGG	89	XM_008264187.2
	R: GGCGTTCTTGAGGCTGGTTCTG		
Erg 2	F: GTGGGGAGCGAGAACAATTA	87	XM_017338174.1
	R: GTTGTCGGTGAATTGGACCT		
Pmp 22	F: AGAGACAGCCATAAGGAGAACG	96	XM_017343068.1
	R: TGCAGACCACACCCTTCAT		
Sox 10	F: TCCAAAAACTAATCACAACAATCG	141	XM_008266894.2
	R: GAAGTGCAATTGGGATGAAAA		

### Cell culture and treatment

The RSC 96 cells (a rat SC line) were purchased from ScienCell Research Laboratories. At three passages, the cells were incubated in high-glucose (HG) environment to imitate pathogenic change in T2DM according to our previous report^40^. In brief, after seeding on a 6-plate well with a density of 5 × 10^5^/mL, SCs were placed into a humidified incubator overnight. Next day, the medium was changed to 30 mM HG medium mixing with/without aFGF (100 ng/ml). Then, the cells were exposed in this concentration of HG condition for another 12 h. We regarded HG plus aFGF as HG + aFGF group. The cells that only cultured in HG medium and normal medium were taken as HG group and control group, respectively.

For the preparation and transfections of small interfering RNA (siRNA), SC lines at 70–80% confluence were infected with 200 pmol Nrf2-siRNA or negative control siRNA (Genechem Co., Ltd., Shanghai) in serum-free medium. 24 h later, cells were cultured in HG + aFGF medium. Cells that were treated with scrambled siRNA control plasmid, together with aFGF and HG medium, were regarded as vehicle group. Specific silencing was identified by Western blotting (the best inhibition of Nrf2 expression was siRNA#1 which was shown in Supplementary Fig. 1). The sequence of Nrf2-siRNA was shown below:

siRNA#1: 5′-CAG AAG AAC UGA UAG AGA U-3′

5′-AUC UCU AUC AGU UCU UCU G-3′

siRNA#2: 5′-GAC AAG GAU GCU CGA GAC U-3′

5′-AGU CUC GAG CAU CCU UGU C-3′

siRNA#3: 5′-GUG ACU UGA AGA CUC UGA U-3′

5′-AUC AGA GUC UUC AAG UCA C-3′.

### Cell migration

The RSC 96 SC lines were seeded in a six-plate well until they were grown to confluency. After serum-starved for 12 h, the cells were scratched manually using a 1 mL plastic pipette tip. Then, each well was washed with PBS to remove unfastened cells and replaced with HG containing with/without aFGF according to the procedure mentioned above. Images of the wound closure or cell migration were taken immediately at time 0 and 12 h after the scratch using PowerShot G10 camera (Canon, Tokyo, Japan). The distance of scratches at 0 h and 12 h was quantified by Image J software and marked as A_0_ and A_12_. The difference between A_12_ and A_0_ in each group was calculated to achieve the migration distance.

### Intracellular ROS detection

The intracellular ROS production was detected using ROS Assay Kit (S0033, Beyotime, China). Briefly, after aspirating off the culture media, cells were incubated with 10 μM 2′, 7′‐dichlorodihydrofluorescein diacetate (DCFH-DA) in serum-free medium for 30 min. Subsequently, the fluorescence images of cells were captured under the Nikon ECLIPSE 80i (Nikon, Japan). Fluorescent intensity was quantified using Image J software.

### Western blotting

Before conducting immunoblotting, we quantified protein concentration in each sample. Except for Nrf2 and Ki67, other biomolecules were extracted for detecting the total protein content. Briefly, homogenized tissue or living cells were lysed with ice-cold RIPA lysis buffer (Beyotime Biotechnology, P0013) containing 1 mmol/L PMSF, and 1 g/mL protease inhibitors. Then, the samples were centrifugated at 16,099*g* for 15 min. The supernatant after centrifuging was used for the immunoblotting. For Nrf2 and Ki67, the whole proteins in cell or tissue were prepared using Nuclear and Cytoplasmic Protein Extraction Kit (Beyotime, Nantong, China). Afterward, the extracted proteins were processed for immunoblotting, including quantification of the protein concentrations, protein separation, transferring onto PVDF membranes, incubating with primary and second antibodies, and signals detection, which were all described previously^41^. The signal intensity of each protein was quantified using Image J software. The relative densities of the bands were normalized to GAPDH or Histone H3 and results were repeated at least three times.

### Immunofluorescence staining

With regard to this experiment, the procedures were fully detailed in the previous description^42^. The tissue sections or cell samples were incubated with the primary antibodies and fluorescent-labeled secondary antibodies in 37 °C condition for 2 h and 30 min, respectively. The image acquisition was visualized with a confocal laser scanning microscope (Leica SP2, Mannheim, Germany).

### TUNEL assay

Cell apoptosis was detected by in situ terminal deoxynucleotidyl transferase-mediated dUTP-biotin nick end-labeling reaction (TUNEL) staining (Roche, 11684817910, Basel, Switzerland). According to the standard protocol, cells were fixed with 4% paraformaldehyde at 4 °C for 20 min. Then, they were incubated with 1 μg/ml proteinase K solution for 15 min. After washing with PBS three times for 15 min, each well was incubated with 100 μL TUNEL reaction mixture for 1 h at 37 °C. Subsequently, cells were stained with 4′, 6-diamidino-2-pheny-lindole (DAPI, Beyotime, Shanghai, China) for 5 min and the images were acquired using a Nikon confocal laser microscope (Nikon, A1 PLUS, Tokyo, Japan).

### Statistical analysis

All data were expressed as means ± standard error of mean (SEM). Statistical comparisons of two or more treatment groups relative to the untreated control were examined by one-way analysis of variance (ANOVA) using the GraphPad Prism software Version 7 (GraphPad Software, Inc, San Diego, CA). For all analyses, a two-sided *P* value of less than 0.05 was deemed statistically significant.

## Results

### Chronic diabetes markedly inhibits aFGF expression in SCs

**Fig. 1:**
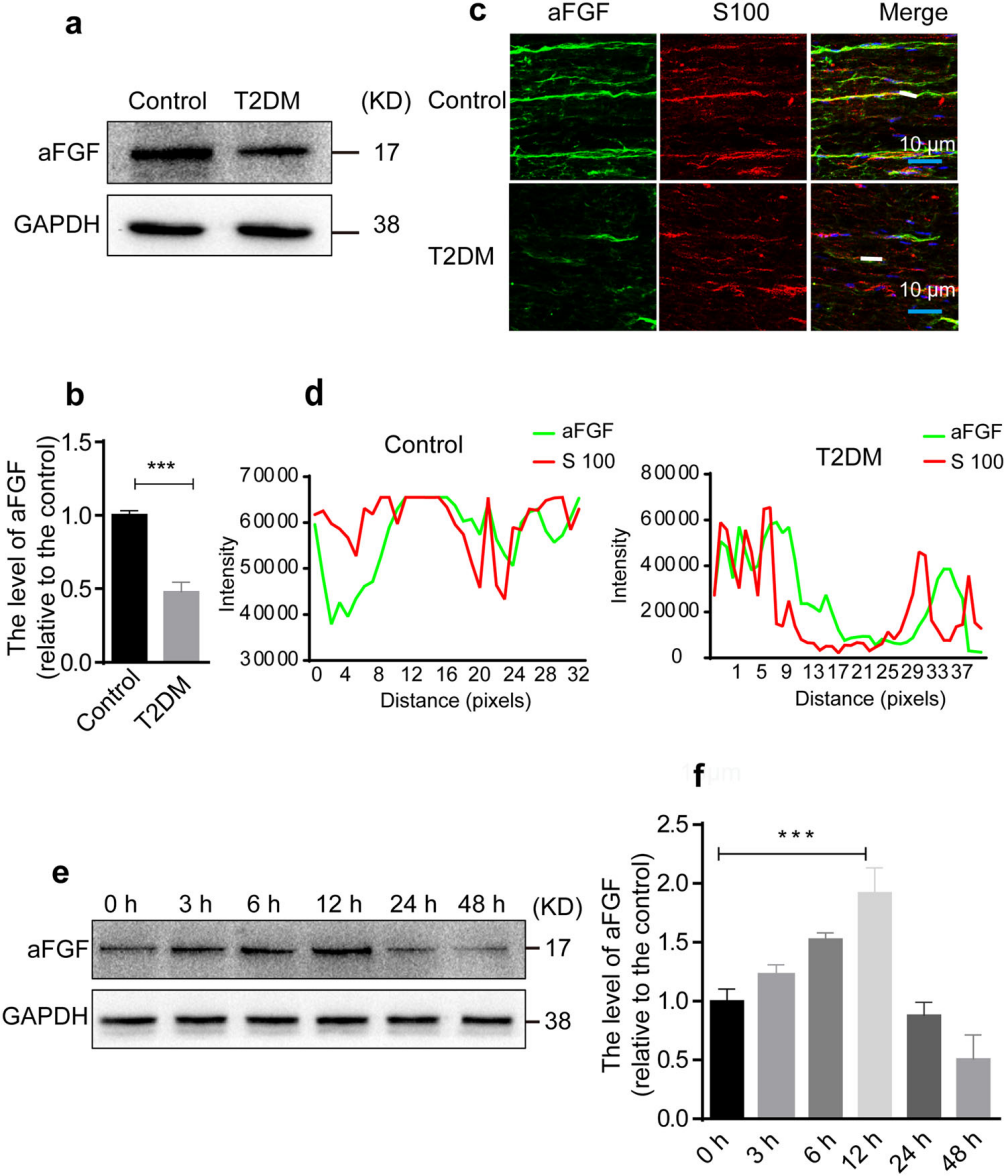
Endogenous levels of aFGF in T2DM mice and SCs. **a**, **b** Western blotting detected the content of aFGF in normal and T2DM mice. The result was repeated at least 3 times. **c**, **d** The longitudinal sections of sciatic nerves from normal and T2DM mice were double-immunostained with aFGF (green) and S100 (red) antibodies. The results of co-localization were analyzed by Image J software and GraphPad Prism. Scale bars represent 10 μm. *n* = 5 mice per group with three images for each mouse. **e**, **f** Immunoblot and quantification data to show the aFGF level in SCs after cultured in HG (30 mM) medium for 3 h, 6 h, 12 h, 24 h, or 48 h. All data are the mean ± SEM of three experiments. ^***^P < 0.001 vs. the T2DM group or the SCs exposing in HG for 12 h.

A reduction of endogenous aFGF was previously implicated in disease progression of T2DM patients^43^. Thus, we tried to detect aFGF level in the sciatic nerve tissues of T2DM mice. Western blotting showed that T2DM mice expressed lower levels of aFGF than control mice (*P* < 0.001; Fig. 1a and b). Furthermore, we also found that the fluorescence intensity for aFGF was nearly colocalized with that for S100 (indicating mature SCs), suggesting that aFGF is mainly expressed in SCs (Fig. 1c and d). Next, we cultured SC lines under high-glucose (HG) conditions (30 mM glucose) in vitro to mimic hyperglycemic conditions for diabetic patients, and measured the changes of aFGF expression at the indicated time points. As the result shown (Fig. 1e and f), the expression of aFGF gradually increased as early as 3 h and peaked at 12 h, after which aFGF level began to decrease. This phenomenon might indicate that SCs themselves secreted a certain amount of aFGF at an early time stage to resist the deteriorating external environment. Whereas, if this deterioration was continued for a prolonged time, SCs would suffer damage or apoptosis, leading to a decrease of aFGF production. In short, these experiments demonstrate that aFGF plays an important role in SCs under hyperglycemic conditions.

### aFGF attenuates diabetes-induced demyelination

**Fig. 2:**
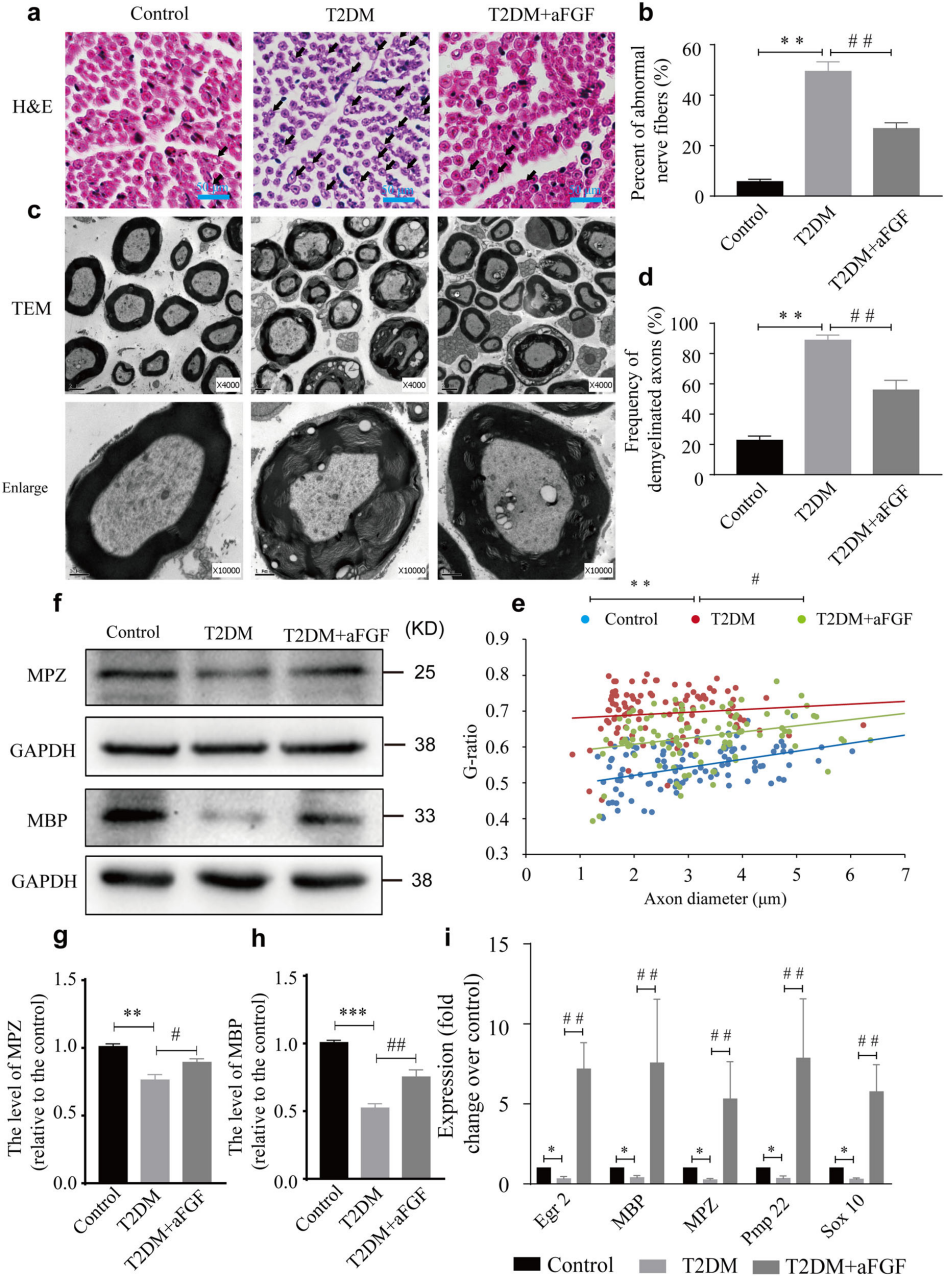
aFGF promotes remyelination in T2DM model. **a**, **b** Histomorphology images of H&E staining from the sciatic nerves in the control, T2DM and T2DM + aFGF groups. The vacuolar-like structure of nerve fibers were indicated by green arrows. Their percent in each group were quantified with Image J software. Scale bar = 50 μm. **c** Representive TEM images in each group. Magnification of ×4000 (scale bar = 2 µm, middle), magnification of ×10,000 (scale bar = 1 µm, bottom). **d**, **e** A bar graph and scatter plot showing the frequency of abnormal myelin and the g-ratio in each group from (**c**). **f–h** Protein expressions of MPZ and MBP for the three experimental groups. **i** The expression of Egr2, MBP, MPZ, Pmp22, and SOX10 from different groups was detected via RT-PCR. Each gene expression value was normalized to β-actin. Data were the mean ± SEM, *n* = 5 animals per group for H&E staining and TEM. For western blotting and RT-PCR, each experiment was independently repeated at least 3 times. ^*^P < 0.05, ^**^P < 0.01, ^***^P < 0.001 vs. the control group; ^#^*P* < 0.05, ^##^*P* < 0.01 vs. the T2DM group.

To determine the therapeutic effect of aFGF on remyelination, we firstly examined the morphological changes in the sciatic nerves of mice in each group using H&E staining and TEM. Histopathological analysis of transverse sections of the nerve stumps in the control group had uniform, ordered, and dense myelination with structural integrity. In T2DM mice, massive nerve fibers exhibited vacuolar-like defects (Fig. 2a, marked with black arrows, indicating abnormal nerve fibers), and the percentage of these abnormal nerve fibers was noticeably more than that in the control group (*P* < 0.01; Fig. 2b). Moreover, morphological observation of the inner myelin in the T2DM group revealed many vacuoles, accompanied by onion bulb-shaped structures (shown in an enlarged image in Fig. 2c, defined as demyelination). In comparison, the axonal demyelination was largely alleviated in the T2DM + aFGF group, which was also confirmed by quantifying the proportion of demyelinated axons and analyzing the G-ratio (T2DM + aFGF vs. T2DM: *P* < 0.01 for abnormal myelin and *P* < 0.05 for G-ratio; Fig. 2d and e). Next, we tested the expression of the myelin-associated proteins, MPZ and MBP, in each group through Western blotting. As illustrated in Fig. 2f–h, T2DM significantly reduced the levels of these proteins, but aFGF treatment appeared to attenuate the reduction of MPZ and MBP that observed in the nerves of T2DM + aFGF group. Similarly, the administration of exogenous aFGF largely activated myelination-related genes, including Egr 2, MBP, MPZ, Pmp 22 and Sox10, compared to the T2DM mice without any treatment (all *P* < 0.01; Fig. 2i). Collectively, these data suggest that aFGF enhances remyelination in T2DM in vivo.

### aFGF facilitates SC proliferation and migration

**Fig. 3:**
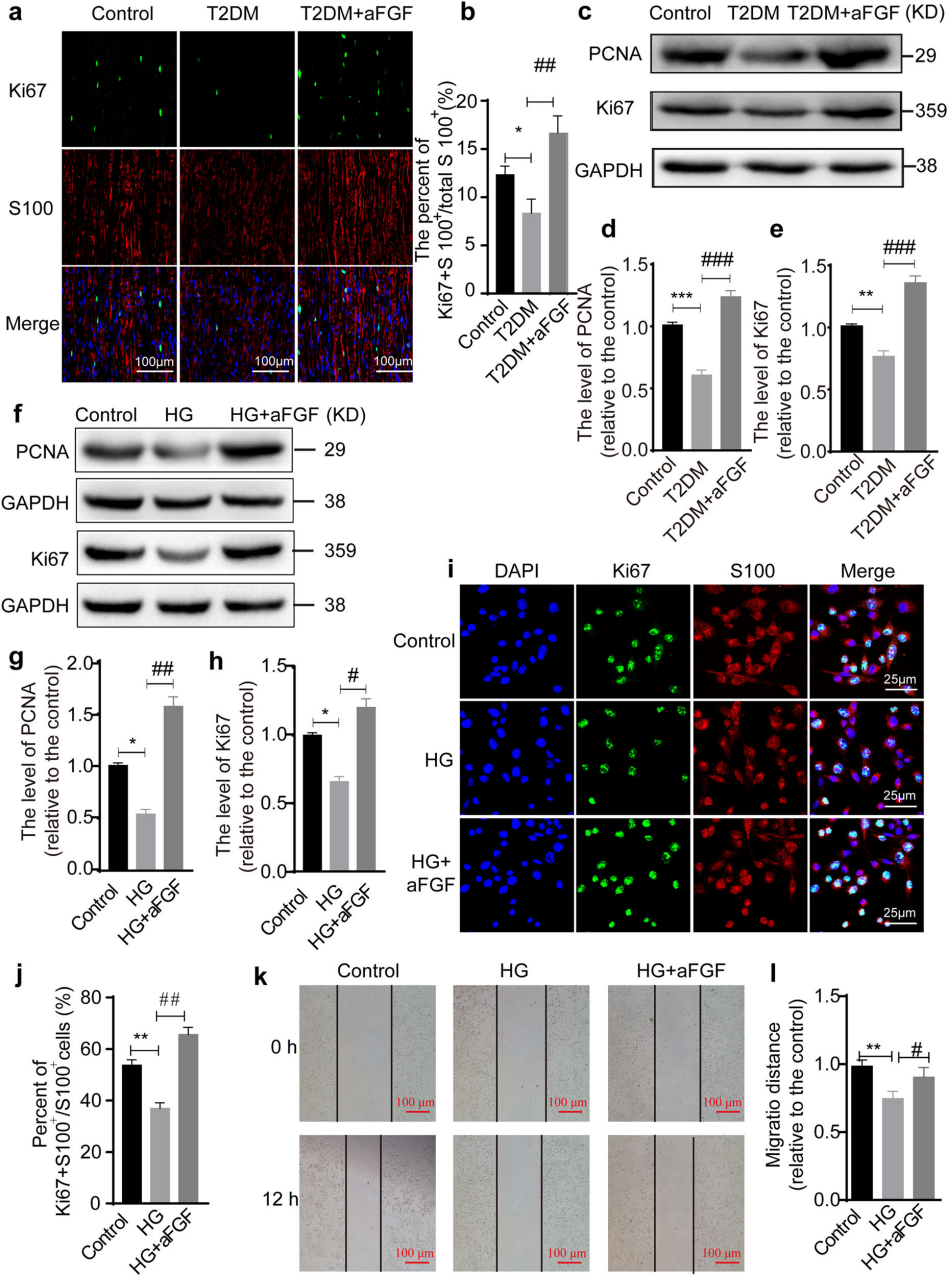
aFGF enhances SCs proliferation and migration. **a** Co-immunofluorescence images showed the distribution of Ki67^+^ (green) labeling nucleus were colocalized with S100 (red) in the control, T2DM and T2DM + aFGF groups. Scale bar equals 100 μm. **b** Quantification of Ki67 positive cells/100 mm^2^ in each group. n = 7 animals per group. **c**-**e** Western blotting analyzed the level of PCNA and Ki67 in each group (*n* = 3). GAPDH was used as an internal loading control and all protein expression was shown as relative levels to that of the control. **f**–**h** The expression of PCNA and Ki67 proteins was quantified in SCs of control, HG and HG + aFGF groups (*n* = 3). **i**–**j** Immunofluorescence staining and quantification data of Ki67 (green) and S100 (red) signals in the cell level. Nucleus was stained with DAPI. Scale bar = 25 μm. **k**, **l** SC migration analysis was detected through a wound-healing assay at 0 and 12 h after scratch. *n* = 3, scale bar = 100 μm. Data are the mean ± SEM. ^*^P < 0.05, ^**^P < 0.01, ^***^P < 0.001 vs. the control group; ^#^*P* < 0.05, ^##^*P* < 0.01, ^###^*P* < 0.001 vs. the T2DM group or HG group.

Substantial evidence has supported the notion that the enhanced proliferation and migration of SCs are a prerequisite for myelin regeneration^44^. To determine this effect in high-fat diet-fed T2DM model, we administered 0.5 mg/kg aFGF to T2DM mice by intraperitoneal injection every other day for 1 month. The degree of SC proliferation in each group was evaluated by immunostaining and immunoblotting. Double-immunostaining for Ki67 (to determine cell proliferation) and S100 (a marker of SCs) showed that Ki67 immunoreactivity was colocalized with the nuclei and distributed within the cytoplasm of S 100-labeled immature SCs in all three groups. Moreover, there were substantially fewer Ki67^+^ cells in T2DM mice than that in the normal mice, but aFGF treatment significantly reversed this trend (*P* < 0.05; Fig. 3a and b). Similarly, immunoblotting also exhibited that the protein levels of Ki67 and PCNA (another biomarker for cell proliferation) in sciatic nerves from T2DM mice were significantly lower than both of the T2DM + aFGF and control groups (all *P* < 0.05; Fig. 3c–e).

Next, we used HG-treated SCs as an in vitro experimental model to evaluate whether aFGF could potentially facilitate their proliferation and migration. After 12 h of treatment, the SC proliferation rate was markedly reduced under HG condition but significantly rescued by aFGF treatment, which was reflected by the value of Ki67^+^ positive cells and quantification of the band densities in each group by immunoblotting (Fig. 3f–j). Through a wound-healing assay, we found that the migratory ability of SCs was markedly impaired under HG conditions for culturing 12 h. However, this inhibition was entirely reversed by aFGF treatment (Fig. 3k and l). All of the above data suggest that aFGF plays a strong capacity to promote SC proliferation and migration.

### aFGF attenuates SC apoptosis

**Fig. 4:**
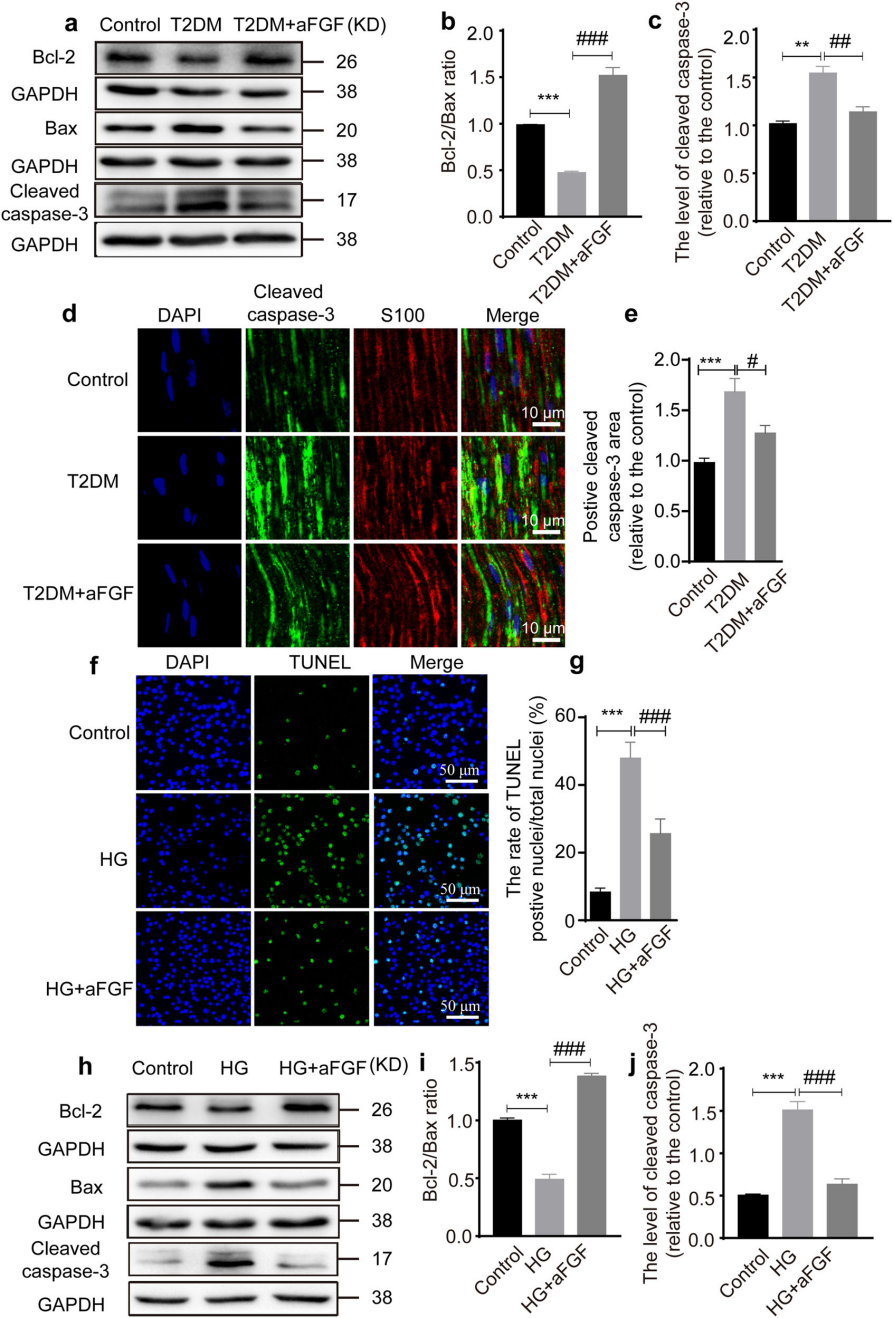
aFGF inhibits SCs apoptosis. **a**–**c** Representative western blotting bands plus the corresponding statistical results of Bax, Bcl-2 and cleaved caspase-3 in control, T2DM and T2DM + aFGF groups. Data were repeated three times. **d** Co-staining of cleaved caspase-3 (green) and S100 (red) in each group. Nucleus were labeled with DAPI (blue). Scale bar equals 10 μm. **e** Quantification of positive cleaved caspase-3 area from (**d**). *n* = 7 animals per group. **f** Representative SC apoptosis images at 12 h after HG. Scale bar = 50 μm. **g** Quantification of the percentage of TUNEL-positive SCs from (**f**). This test was independently repeated 5 times. **h** Protein level of Bax, Bcl-2 and cleaved caspase-3 among the Control, HG, HG + aFGF groups. **i**, **j** Densitometric analyses of Western blotting from (**h**). This experiment was repeated in triplicate. Data are the mean ± SEM, ^**^P < 0.01, ^***^P < 0.001 vs. the control group; ^#^*P* < 0.05, ^##^*P* < 0.01, ^###^*P* < 0.001 vs. the T2DM group or HG group.

To assess whether aFGF treatment promotes SC viability under hyperglycemic condition, we detected the apoptosis-related proteins via immunoblotting. As illustrated in Fig. 4a–c, the protein levels of cleaved caspase-3 in the sciatic nerve was significantly induced by T2DM. Whereas, the ratio of Bcl-2/Bax in T2DM exhibited reverse. In comparison, aFGF treatment largely reversed these changes (the statistical significance of differences of both the Bcl-2/Bax ratio and cleaved caspase-3 level between the T2DM + aFGF vs. T2DM groups was *P* < 0.05.). Double-immunostaining result revealed that the cleaved caspase-3 signal was nearly contained within the S100-positive SCs in all experimental groups (Fig. 4d). Moreover, the positive signal for cleaved caspase-3 in each group was consistent with the results indicated by immunoblotting (Fig. 4e).

An in vitro apoptosis assay showed that there were significantly more TUNEL-positive nuclei in the HG group comparable to that in the control group (*P* < 0.001; Fig. 4f and g), but this increase was largely reduced after aFGF treatment (HG + aFGF vs. HG: *P* < 0.001). Similarly, changes in expression of these apoptotic-related proteins in SCs were consistent with those in vivo (Fig. 4h–j). These results further demonstrate that aFGF attenuates the apoptosis of SCs exposed to HG condition.

### aFGF treatment reduces hyperglycemia-induced oxidative stress in SCs

**Fig. 5:**
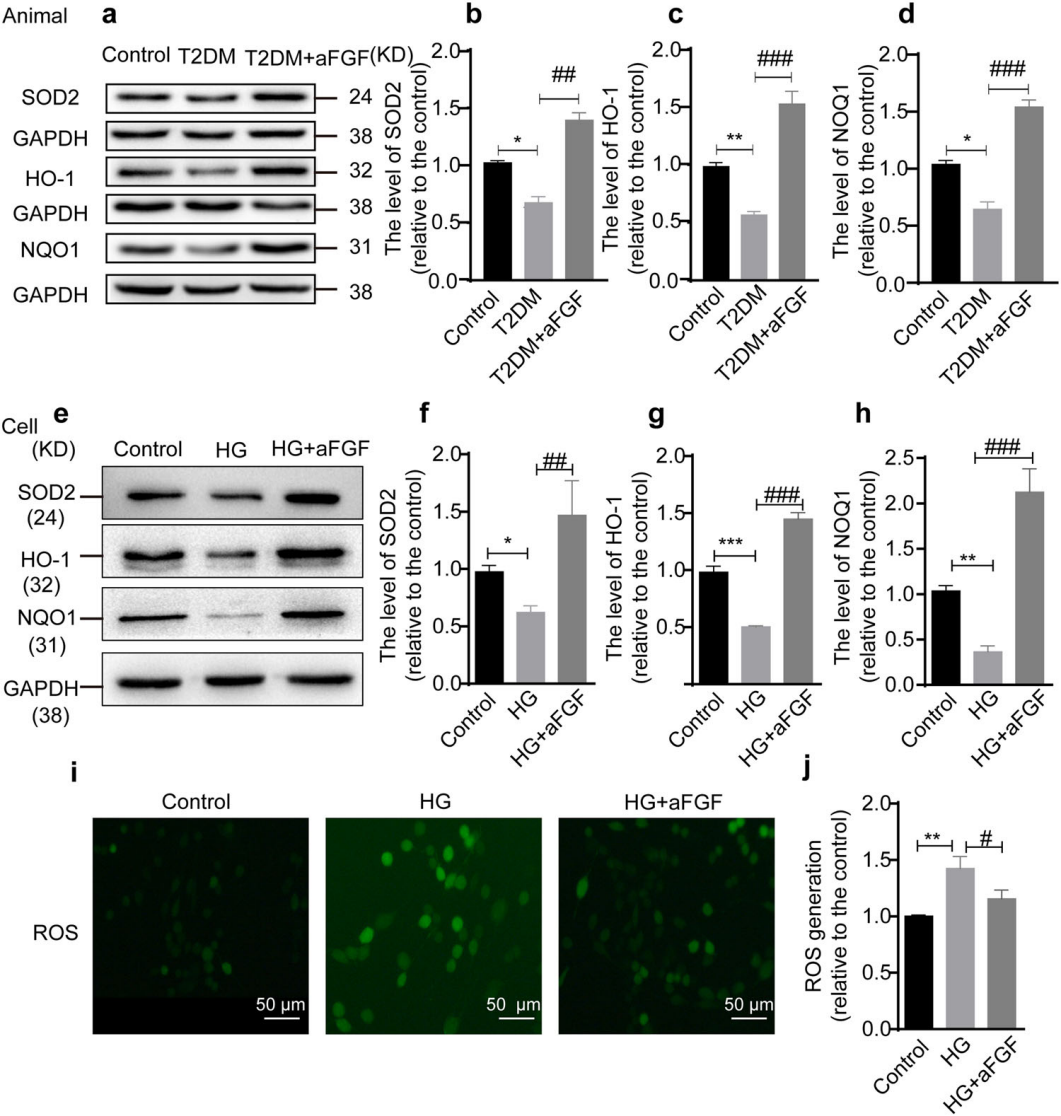
aFGF weakens the oxidative damage and facilitates antioxidant capacity. **a**, **d** The representative band of immunoblotting and quantification analysis of SOD2, HO-1 and NQO1 proteins level among the Control, T2MD, T2MD + aFGF groups. This experiment was repeated in triplicate. **e**-**h** Western blotting analysis of SOD2, HO-1, and NQO1 in SCs with HG in the absence or presence of aFGF. The relative quantification of SOD2, HO-1, and NQO1 was quantified from e (n = 3). **i**-**j** ROS assay kit detected the expression of reactive oxygen species in SCs among the three groups. This teat was repeated in triplicate. Scale bar = 50 μm. All data are the mean ± SEM. ^*^P < 0.05, ^**^P < 0.01 ^***^P < 0.001 vs. the control group; ^#^*P* < 0.05, ^##^*P* < 0.01, ^###^*P* < 0.001 vs. the T2DM group or HG group.

Prolonged hyperglycemia is responsible for inducing oxidative stress as evident by increased ROS production and decreased antioxidant proteins, including SOD2, HO-1 and NQO1^45,46^. Here, we determined whether the aFGF administration could reverse hyperglycemia-induced oxidative stress activation. Compared with the control mice, the protein expressions of SOD2, HO-1, and NQO1 were significantly reduced in the sciatic nerve tissues of T2DM mice. However, aFGF treatment greatly reversed these changes (Fig. 5a–d).

Next, we compared the change of these proteins in control, HG and HG + aFGF groups. Consistent with the results in the animal model, the decreasing expression of SOD2, HO-1 and NQO1 in HG-cultured SCs were dramatically reversed after supplementation of the culture medium containing aFGF (Fig. 5e–h). In addition, ROS production was evaluated through DCFH-DA detection, a typical method used to measure ROS. As shown in Fig. 5i, j, a lower level of ROS production was detected in HG + aFGF group, when compared with the HG group. Collectively, these data indicate that aFGF has able to prevent hyperglycemia-induced oxidative stress activation both in vivo and in vitro.

### aFGF prevents hyperglycemia-induced oxidative stress through activating keap1/Nrf2 signaling

**Fig. 6:**
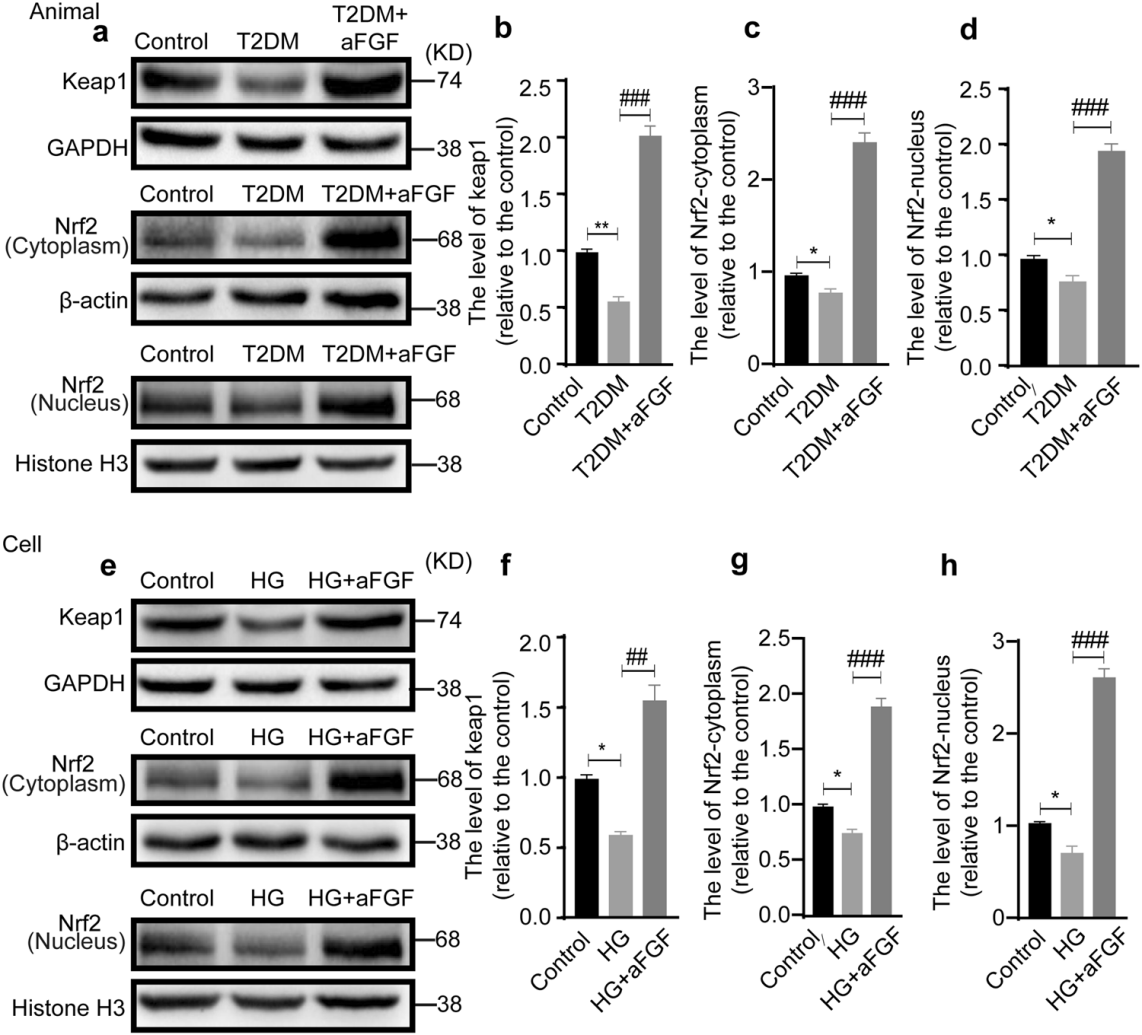
The antioxidant capacity of aFGF is associated with activating the Keap1/ Nrf2 signal. **a** Western blotting analysis showing the protein level of Keap1 and Nrf2 separated from the cytoplasm and nucleus in the sciatic nerves of T2DM model treating with/without aFGF. **b**, **d** Densitometric analyses of these proteins from (**a**). **e** Representative Western blotting of Keap1, cytoplasmic Nrf2, and nuclear Nrf2 in the control, HG and HG + aFGF groups. **f**–**h** Quantification of these proteins from (**e**). All data were normalized to GAPDH or Histone H3 and showed as relative levels to that of the control. Moreover, all data are the mean ± SEM (n = 3). ^*^P < 0.05, ^**^P < 0.01 vs. the control group; ^##^*P* < 0.01, ^###^*P* < 0.001 vs. the T2DM group or HG group.

Growing evidence has been shown that, under stress condition, such as chronic hyperglycemia, Keap1/Nrf2 signaling played a pivotal role in regulating the antioxidant response^47,48^. Here, we found that the expression of Nrf2 in the cytoplasm and nucleus and Keap1 were significantly downregulated in T2DM mice but were greatly elevated after aFGF treatment (Fig. 6a–d). Similar to the results in vivo, western blotting also revealed that the addition of aFGF significantly enhanced the intensities of the bands for Nrf2 and Keap1, compared with those in the HG group (Fig. 6e–h). Overall, our current results suggest that aFGF treatment significantly increases Keap1/Nrf2 signaling activation.

### Silencing Nrf2 gene significantly abolishes aFGF biological effect on protecting SCs from HG-induced stress and apoptosis

**Fig. 7:**
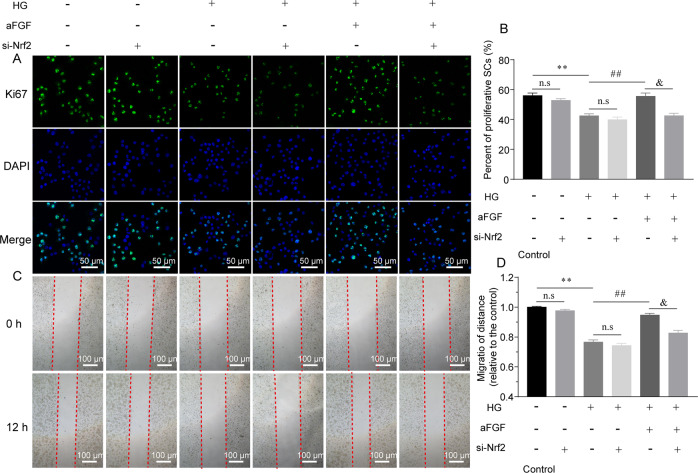
Nrf2 signaling blockade in the HG condition had significant effect on inhibiting aFGF-medicated SC proliferation and migration. **a**, **b** Representative images and quantitative analysis of immunostaining for Ki67 (green) in different treatment groups. Scale bar = 50 μm. **c**, **d** Representative wound-healing images and quantification data of SCs at 0 h and 12 h after treatment. Scale bar = 100 μm. Data are the mean ± SEM from three independent experiments. n.s indicated not significant difference between the control and control+si-Nrf2 groups, and between the HG and HG + si-Nrf2 groups; ^**^P < 0.01 vs. the control group; ^##^*P* < 0.001 vs. the HG + aFGF group; ^&^*P* < 0.05 vs. the HG + aFGF+si-Nrf2 group; n.s indicated not significant difference between compared two groups.

**Fig. 8:**
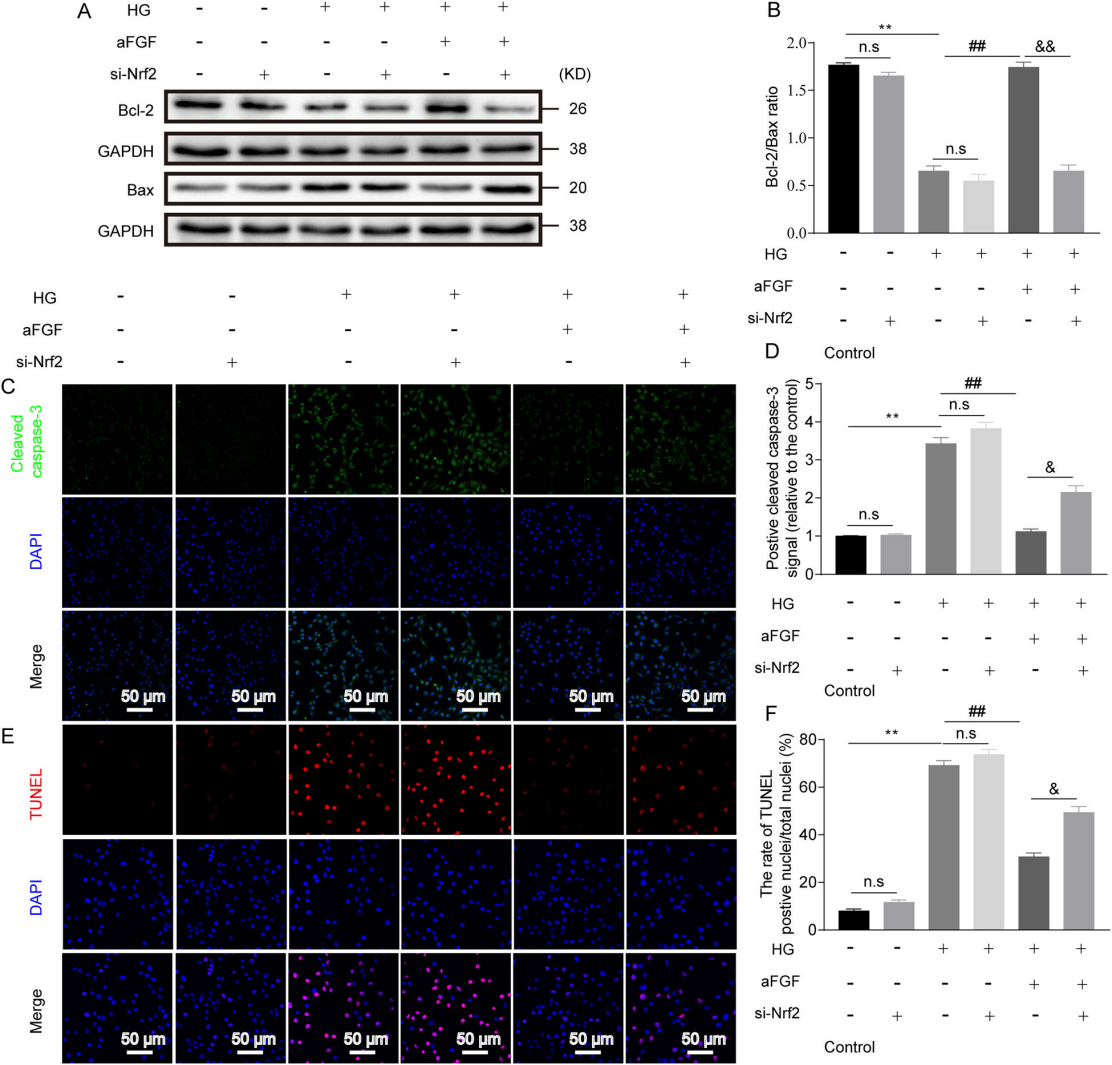
Inactivation of Nrf2 signaling aggravated HG-induced SC apoptosis under the condition of aFGF treatment. RSC 96 cells were incubated with si-Nrf2 for 24 h. Then, the medium was changed and cells were cultured in control, HG or HG + aFGF condition for another 12 h. The apoptotic extent was determined by various detecting methods. (**a-b**) Representative immunoblotting images of Bcl-2 and Bax and their ratio in each group. (**c-d**) Representative immunostaining images and immunoreactivity of Cleaved caspase-3 in each group. Nuclei are counterstained with DAPI (blue). Scale bar = 50 μm. (**e-f**) Apoptosis in SCs by TUNEL staining from the six groups and quantification of the rate of TUNEL-positive nuclei/total nuclei. Nuclei are counterstained with DAPI (blue). Scale bar = 50 μm. All data are expressed as the mean ± SEM from three independent experiments. ^**^P < 0.01 vs. HG group; ^##^*P* < 0.01 vs. the aFGF group; ^&&^*P* < 0.001 vs. the aFGF+Si-Nrf2 group; n.s indicated not significant difference between compared two groups.

To further determine the important role of aFGF-medicated Keap1/Nrf2 axis in protecting SC behavior, we silenced Nrf2 gene expression with the conditions of control, HG, HG + aFGF via transfecting Nrf2 siRNA into SCs. After 24 h transfection, there occurred a significant decrease of Ki67^+^ positive cells and migration distance (Fig. 7), but increase of Bcl-2/Bax ratio, Cleaved caspase-3 expression and TUNEL-positive cells (Fig. 8). In contrast, silencing the Nrf2 gene had no significant altered SC behavior with the conditions of control and HG (Figs. 7, 8).

In parallel, we also downregulated Nrf2 expression by using a novel and specific Nrf2 inhibitor, ML385 (5 μM)^49^, prior to culture in HG medium containing aFGF. Similar to Nrf2 siRNA, ML385-treated cultures manifested a higher ROS generation (Fig. S2a, b) and cellular apoptosis (Fig. S2l–p) compared to only HG + aFGF treating SCs. Whereas, immunoblotting and the scratch test revealed that the expression of antioxidant and proliferation-related proteins, as well as the speed of SC migration exhibited a reversed trend, i.e., the HG + aFGF+ML385 group had a lower level of antioxidant and proliferation-related proteins (Fig. S2c–i) and slower speed of SC migration (Fig. S2j, k) than the HG + aFGF group. Hence, aFGF-medicated oxidative stress suppression and cellular behavior amelioration under HG condition are probably controlled by activating the Keap1/Nrf2 pathway in vitro.

## Discussion

The new findings of the current study were as follows: (1) aFGF has a potential therapeutic effect in ameliorating myelin pathology and increasing the expression of myelin-related genes in a prolonged T2DM model, (2) this beneficial effect is probably due to the aFGF-mediated increase in SC proliferation and migration and simultaneously attenuation of SC apoptosis, and (3) we determined that the molecular mechanism underlying aFGF controlling myelination and SC deficits under hyperglycemic conditions might be related to the Nrf2/Keap1 signaling-mediated suppression of oxidative stress. These findings highlight aFGF, as an available therapeutic strategy, ameliorates the pathological change of demyelination during the pathogenesis of T2DM and reveal its underlying molecular mechanism.

It has been shown that long-term exposure to elevated level of glucose would give rise to chronic demyelination, a notable characteristic of neuropathy that seriously damaged myelin, axons, and neuron cell bodies. In the PNS, axonal demyelination is closely associated with SC abnormalities, because myelin formation depends on SC development and maturation to wrap around axon. Besides, proliferating SCs have capable of providing abundant neurotrophins and oxygen to supporting axonal regeneration. Under diabetic condition, SCs are vulnerable to glucotoxicity-induced damage or apoptosis, which deteriorates myelin splitting and myelin shedding, leading to axonal dysfunction and demyelination. Previous reports have shown that hyperglycemia-induced SC dysfunction and toxicity were associated with metabolic alterations and/or inflammatory aggravation^50,51^. Increasing evidence has focused on altering SC behavior to reverse progressive myelin degeneration. For instance, converting the SC phenotype from a denervated state to an elongated and bipolar morphology promotes the shortening of remyelination^52^. Overexpression of Neuregulin-1 type I (NRG1-I) was found to be required for SC differentiation, which further accelerates remyelination in chronic demyelinating neuropathy^53^. Moreover, SC proliferation and dedifferentiation are requisite for myelin generation and maintenance^54^. Therefore, we try to restore myelin disorganization and maintain normal myelin morphology through improving SC plasticity and activating intrinsic reprogramming in SCs.

aFGF plays multiple regulatory roles in the biological processes of cellular survival, proliferation, migration, and plasticity. Previous our and the others’ works had confirmed that aFGF possessed striking neuroprotective and neuroregenerative effects in the nervous system^55,56^. Increasing evidence has indicated that aFGF could prevent the occurrence and development of a number of diabetic complication, including diabetic cardiomyopathy, hepatic damage, and nephropathy^57–59^. More importantly, Suh. *et al* first reported that only a single injection of recombinant aFGF provoked a sustained decrease in glucose and insulin sensitization in diabetic hyperglycemia^23^. This exciting discovery was also verified in another report that utilized a common aFGF at 1/10 the dose to treat a rodent model of T2DM^22^. Accordingly to these reports, we speculate that aFGF plays a positive role in improving SC/myelin pathology in diabetic hyperglycemia.

To verify this hypothesis, high-fat diet-fed T2DM mice and SCs exposed to high glucose were treated with/without aFGF. At predetermined times, pathology parameters in the sciatic nerve or SCs were assessed. As expected, severe demyelination and SC apoptosis in the T2DM model was dramatically reversed after aFGF administration. Simultaneously, SC proliferation and migration increased as well. These data indicated that the injection of aFGF reduced demyelination and increased the expression level of myelin-associated proteins, which was critically related to aFGF-induced enhancement of survival, proliferation and migration in SCs. Nevertheless, the potential mechanism that regulates the effect of aFGF in ameliorating progressive demyelination in this condition remains unknown.

Recent evidence has demonstrated that increased oxidative stress and ROS are the leading causative factors driving the pathogenesis of T2DM^60–62^. During the development of T2DM, the cells and tissue of the body are exposed to hyperglycemic condition. Such elevated glucose levels promote progressive oxidative stress activation, resulting in excess ROS production. The overproduction of ROS can alter cell destiny and impair the structure and function of body tissues, such as the peripheral nerve^63^. Hamilton. *et al* reported that oxidative stress was closely associated with the reduced myelination in peripheral neuropathies^31^. Moreover, accumulating evidence has shown that oxidative stress could reduce SC survival and induce SC apoptosis^64,65^. However, we still unknown whether the beneficial effect of aFGF in preventing myelin pathology and protecting SCs from HG-induced apoptosis was related to excessive oxidative stress activation. Presently, we documented that the sciatic nerves in T2DM mice expressed low levels of the anti-oxidative related proteins, including SOD2, HO-1, and NOQ1, but aFGF significantly reversed this trend. Furthermore, the results of an in vitro experiment showing that aFGF regulated SC behavior under high-glucose conditions was consistent with the in vivo results. These data indicate that the aFGF-induced reversal of SC dysfunction and demyelination may be associated with its suppression of excessive oxidative stress activation.

The Keap1/Nrf2 pathway is regarded as the primary mediator of oxidative stress responses in a wide array of disease models^66^. It is well known that Nrf2 can be dissociated from the Keap1/Nrf2 complex upon oxidative stress and translocates into the cell nucleus to induce the transcription of a serious of antioxidant defense genes that contribute to the resistance against excitotoxic and oxidative insults in neurological disorders^67–69^. Activation of the Nrf2 signaling cascade reaction via antioxidant substances is also found to attenuate H_2_O_2_-induced mitochondrial dysfunction and SC apoptosis^70^. Moreover, we previously revealed that a reduction in peripheral nerve injury (PNI)-induced oxidative damage could be achieved through fibroblast growth factor 21 (FGF21)-mediated Nrf2 signaling activation^64^. These findings indicate that Keap1/Nrf2 signaling may be the main upstream regulator that controls oxidative stress-induced demyelination in T2DM. To confirm this hypothesis, the protein expression of Nrf2 and Keap 1 in each group was detected using immunoblotting. The results showed that the decrease in Nrf2 and Keap1 in the T2DM model was reversed after direct treatment with aFGF. These findings were consistent with our results collected from a SC line cultured under high-glucose condition. Additionally, the suppression of Nrf2 activation through genetic or pharmacological methods in vitro significantly restored oxidative stress, resulting in the decrease of SC survival. These results suggest that the Keap1/Nrf2 axis maybe act as the underlying mechanism for guiding aFGF to attenuate excessive oxidative damage in SCs.

Nevertheless, aFGF-mediated SC protection and neuroplasticity should also be regulated by other signaling cascades. For instance, endogenous aFGF has been shown to specifically express in motor and sensory neurons, which was participated in neurite outgrowth and axonal regeneration via activating MEK-ERK1/2-STAT3-Egr1 pathway after sciatic nerve damage^21,71^. In vitro, the usage of aFGF was required to facilitate proliferation of primary SCs predominantly through JAK/STAT3 transduction pathway^72^. Likewise, aFGF-dependent Rho-GTPase suppression also plays an essential role in decreasing the growth inhibitory molecules, and therefore enhancing intrinsic ability of regeneration in the injured spinal cord^73^. Thus, we speculate all of these underlying mechanisms mentioned above are also participate in aFGF benefit effects on exerting remyelination in T2DM.

Another important issue is selecting the suitable time period of SCs culturing in HG in vitro that matches the similar characteristics of SCs in vivo hyperglycemia condition. In our studies, we observed that the aFGF expression was upregulation at early time and subsequently became downregulation at later time. This expressing change is consistent with the findings of diabetes-induced alterations in the expression of aFGF in vivo model^58,74^. Thus, as for the prolonged T2DM mice, we detected the aFGF expression in their tissues was less than that of the normal mice. The reason is that SCs are susceptible to HG infiltration. If SCs exposing in the HG in vitro is not too long, they triggered a serious of pathological signaling events, including increased ROS levels, weak antioxidant capacity, and depolarization of mitochondria, which manifested the similar characteristics of SCs in vivo hyperglycemia condition^75,76^. Thus, during this period, SCs cultured in HG are suitable for modeling hyperglycemia in T2DM. However, if culturing in HG is too long, SCs display acute metabolic disorder and dysfunction, leading to their death or disintegration^77^. This situation is not only inappropriate to model hyperglycemia in T2DM, but also totally unsuited for the experimental progression. But it is should be noted that the specific time point that distinguishes the physiological state of SCs culturing in HG in vitro from exposing in chronic hyperglycemia in vivo is still unknown. This issue needs to be explored further.

In summary, this study demonstrated that the long-term administration of aFGF facilitated a marked increase in SC proliferation and migration, and suppressed SC apoptosis to ameliorate disordered demyelination in T2DM mouse model. Furthermore, we also revealed that the molecular mechanisms of these beneficial effects were related to the aFGF-mediated excessive oxidative stress blockade, which was seemed to regulate via the activation of the Keap1/Nrf2 signaling (a schematic diagram is shown in Fig. S3). All of these findings indicate that aFGF is a promising therapeutic agent for preventing the progression of chronic demyelinating diseases via reducing diabetes-induced cellular stress.

## Supplementary information

Supplement materials

Supplement figure 1

Supplement figure 2

Supplement figure 3

## Data Availability

The datasets used and/or analyzed during the current study are available from the corresponding author on reasonable request.
