# Supplementary material for: Acidic fibroblast growth factor attenuates type 2 diabetes-induced demyelination via suppressing oxidative stress damage
Source: Cell Death Dis. 2021 Jan 21;12(1):107. doi: 10.1038/s41419-021-03407-2 (PMC7819983; doi:10.1038/s41419-021-03407-2)
Supplement: Supplementary file 1 — Supplement materials [file 41419_2021_3407_MOESM1_ESM.docx]

**Supplemental Materials**

**Acidic fibroblast growth factor attenuates type 2 diabetes-induced demyelination via suppressing oxidative stress damage**

Rui Li ^1,2,3†^, Beini Wang ^1†^, Chengbiao Wu ^2†^, Duohui Li ^1†^, Yanqing Wu ^1^, Libing Ye ^1^, Luxia Ye ^1^, Xiongjian Chen ^1^, Peifeng Li ^1^, Yuan Yuan ^1^, Hongyu Zhang ^1^, Ling Xie ^1^, Xiaokun Li ^1^, Jian Xiao^1*^, Jian Wang ^1*^

^1^ Department of Hand Surgery and Peripheral Neurosurgery, The First Affiliated Hospital and School of Pharmaceutical Sciences, Wenzhou Medical University, Wenzhou, Zhejiang, 325000, China

^2^ Research Center, Affiliated Xiangshang Hospital, Wenzhou Medical University, Ningbo, Zhejiang, 315700, China

^3^ School of Chemistry, Sun Yat-sen University, Guangzhou, Guangdong 510275, China

^†^ These authors contribute equally to this work

**Correspondence**

**^*^** Jian Xiao, School of Pharmaceutical Science, Wenzhou Medical University, Wenzhou,

Zhejiang, China. Email: xfxj2000@126.com;

^*^ Jian Wang, Department of Hand Surgery and Peripheral Neurosurgery,

The First Affiliated Hospital of Wenzhou Medical University, Wenzhou, Zhejiang,

China. Email: jianwang0516@126.com

**Supplement figure legend**

**Figure S1.** Selection of the optimal Nrf2 specific siRNA from constructed samples. RSC 96 cells were incubated with siRNA#1, siRNA#2, siRNA#3 or negative control siRNA, and then, after 24 h, cells were treated with HG+aFGF medium for 12 h. The protein level of Nrf2 in cytoplasm and nucleus were determined by immunoblotting with specific antibodies. (**a**) Relative protein level was expressed as fold increases normalized to the vehicle. (**b** and **c**) β-actin or Histone 3 was analyzed as internal control. All data are expressed as the mean ± SEM from three independent experiments. ^*^*P* < 0.05, ^**^*P* < 0.01, ^***^*P* < 0.001.

**Figure S2. Inhibition of Nrf2 expression suppressed antioxidant capacity and SC survival. a**-**c** ROS generation and quantification in HG+aFGF and HG+aFGF+ML385 groups using ROS Assay Kit. Scale bar = 50 μm. **c-f** Western blotting analysis of the expression of antioxidant-related proteins in both groups. **g-i** Proliferation-related proteins, including PCNA and Ki67, were quantified using immunoblotting. **j-k** SC migration at 0 h and 12 h in different groups was determined by the scratch test. Scale bar = 200 μm. **L-n** The apoptotic proteins (Bax, Bcl-2 and Cleaved caspase-3) in the both groups were detected using western blots. **o**-**p** Representative co-immunofluorescence images of Cleaved caspase-3 (green) and S-100 (red) in each group. Fluorescence intensity of Cleaved caspase-3 was quantified by the integrated density of pixels. Scale bar = 50 μm. Data are the mean ± SEM from two independent experiments. ^&^*P* < 0.05, ^&&^*P* < 0.01 vs. the HG+aFGF+ML385 group.

**Figure S3. Schematic showing the potential protection mechanism of aFGF against diabetic demyelination.** Exogenous aFGF treatment suppressed hyperglycemia-induced excessive activation of oxidative stress via Keap1/Nfr2 signaling, which facilitated SC proliferation and migration, as well as prevented its apoptosis. These serial regulating progress ultimately ameliorated myelin regeneration and functional protein expression in sciatic nerve of T2MD mice.
