# Supplementary figures and images for: Acidic fibroblast growth factor attenuates type 2 diabetes-induced demyelination via suppressing oxidative stress damage
Source: Cell Death Dis. 2021 Jan 21;12(1):107. doi: 10.1038/s41419-021-03407-2 (PMC7819983; doi:10.1038/s41419-021-03407-2)

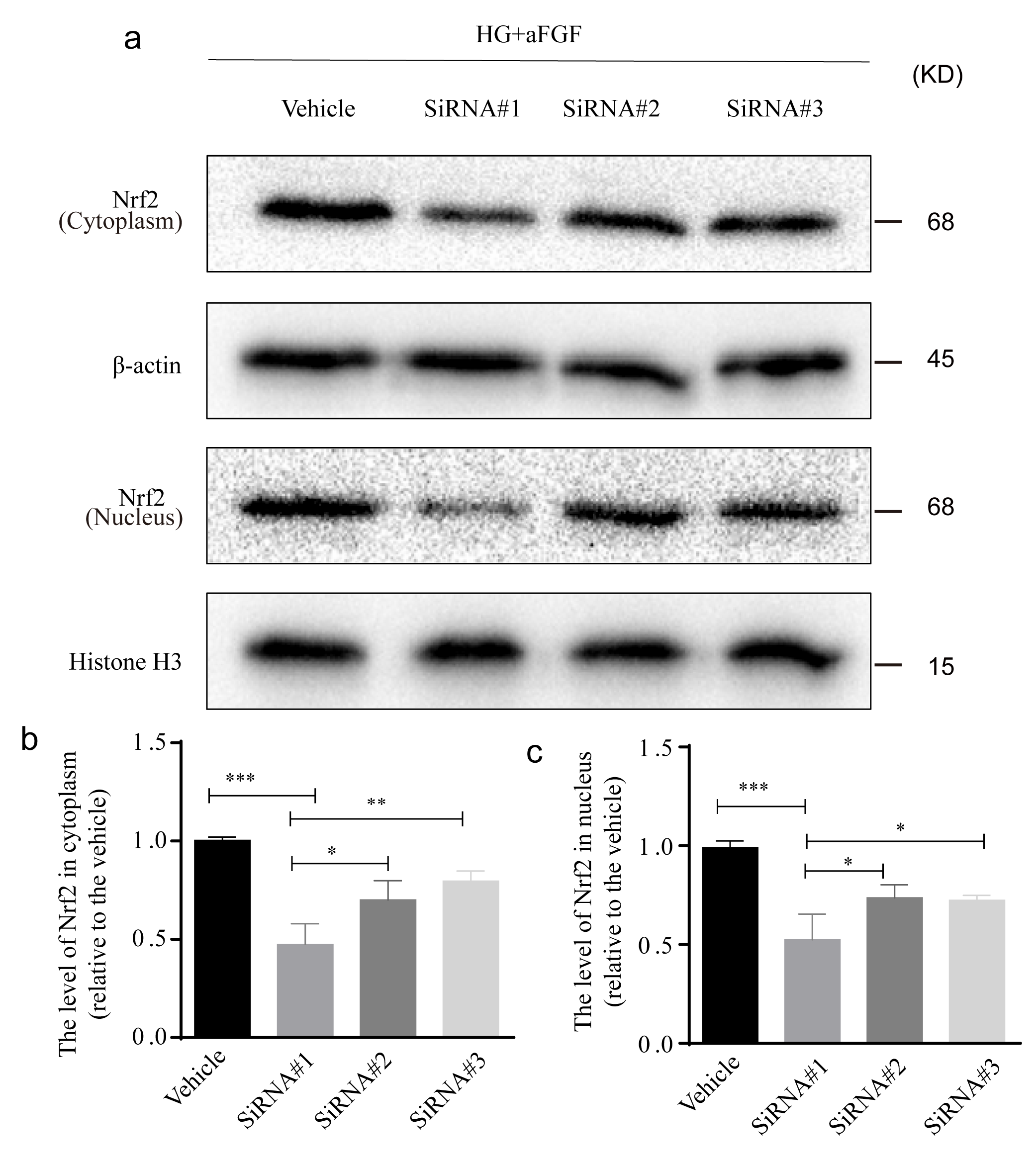

Supplement: Supplementary file 2 — Supplement figure 1 [file 41419_2021_3407_MOESM2_ESM.tif]

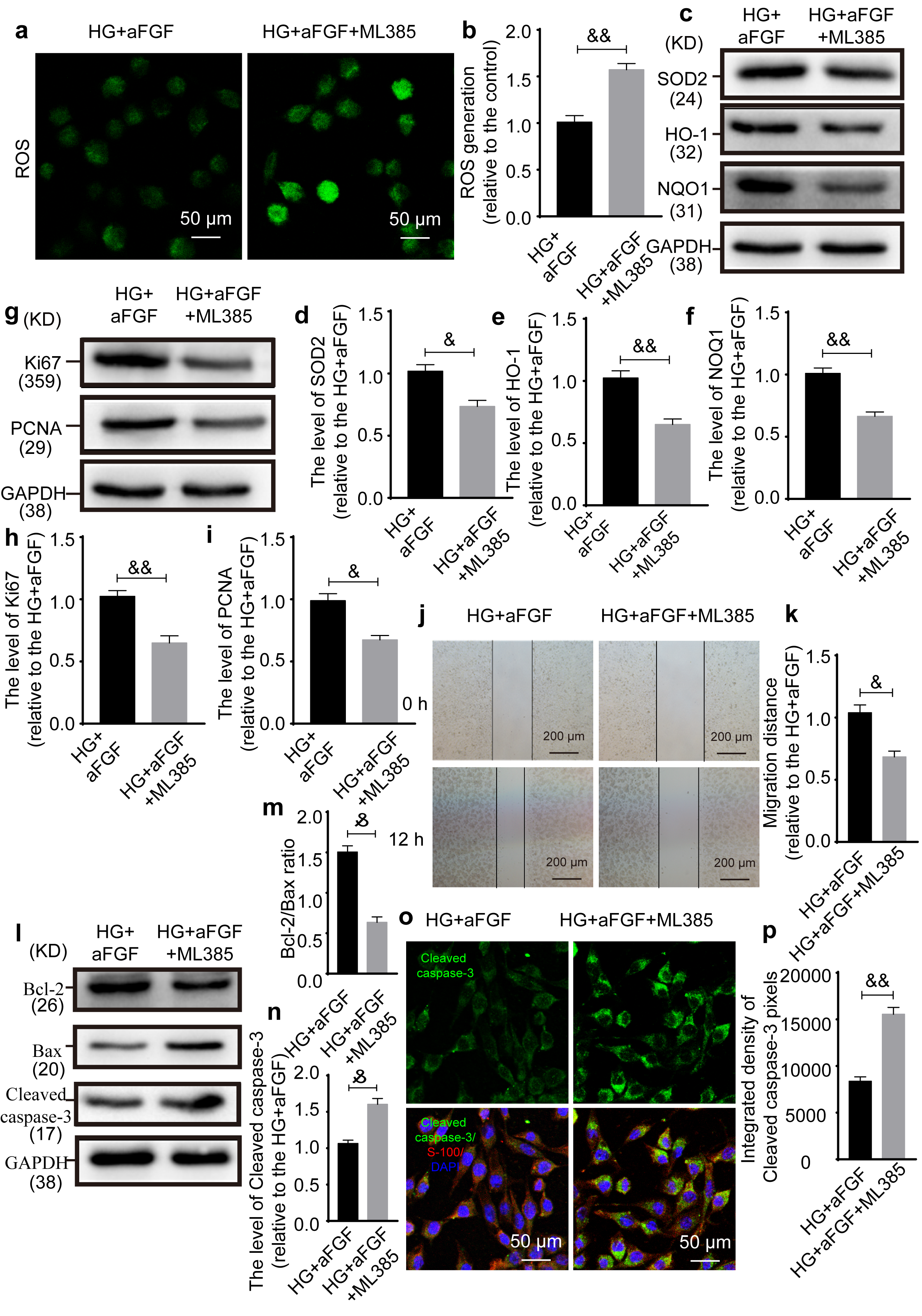

Supplement: Supplementary file 3 — Supplement figure 2 [file 41419_2021_3407_MOESM3_ESM.tif]

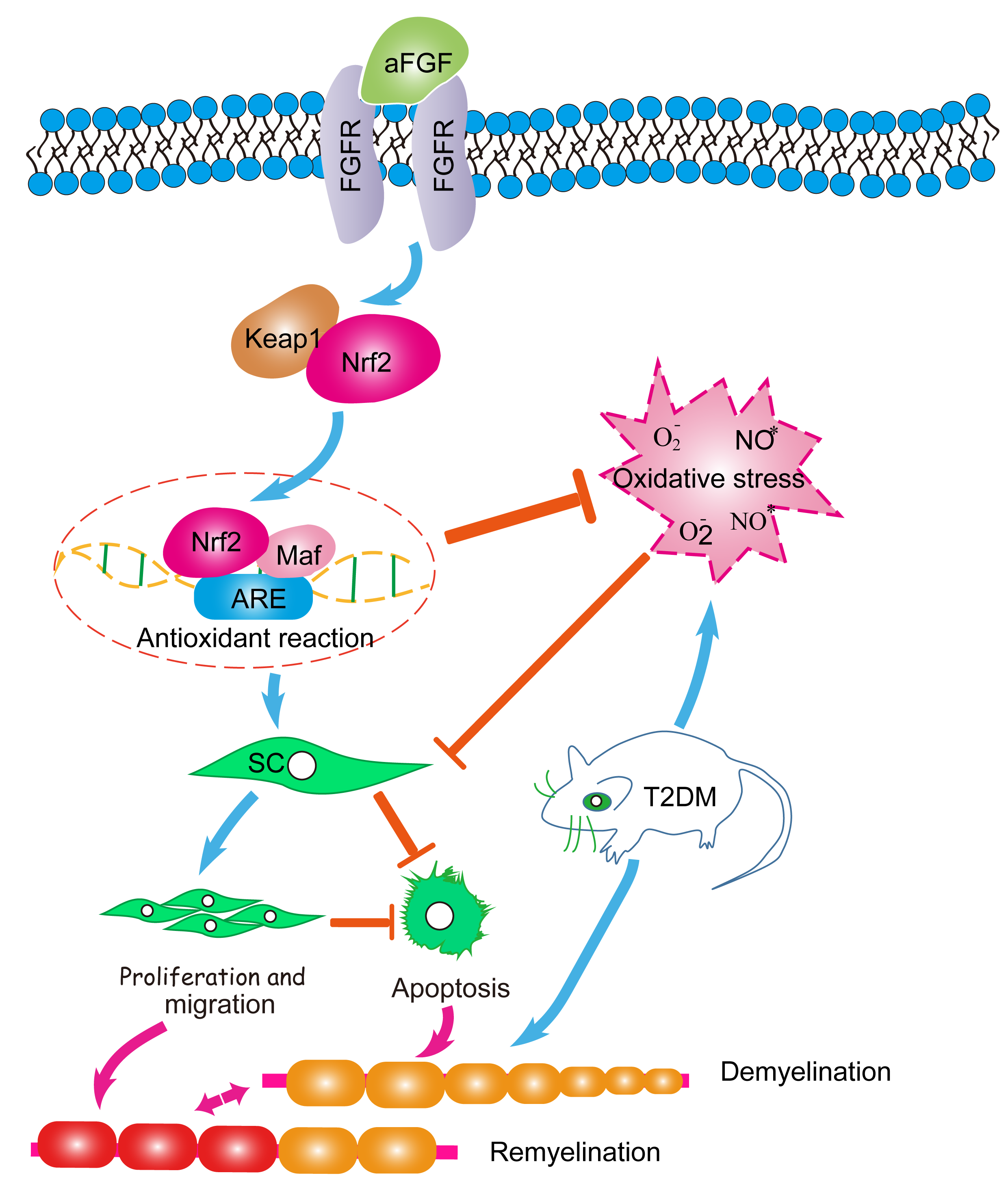

Supplement: Supplementary file 4 — Supplement figure 3 [file 41419_2021_3407_MOESM4_ESM.tif]
